# Profiling Circulating MicroRNA Expression in Experimental Sepsis Using Cecal Ligation and Puncture

**DOI:** 10.1371/journal.pone.0077936

**Published:** 2013-10-30

**Authors:** Shao-Chun Wu, Johnson Chia-Shen Yang, Cheng-Shyuan Rau, Yi-Chun Chen, Tsu-Hsiang Lu, Ming-Wei Lin, Siou-Ling Tzeng, Yi-Chan Wu, Chia-Jung Wu, Ching-Hua Hsieh

**Affiliations:** 1 Department of Anesthesiology, Kaohsiung Chang Gung Memorial Hospital and Chang Gung University College of Medicine, Kaohsiung, Taiwan; 2 Department of Plastic and Reconstructive Surgery, Kaohsiung Chang Gung Memorial Hospital and Chang Gung University College of Medicine, Kaohsiung, Taiwan; 3 Department of Neurosurgery, Kaohsiung Chang Gung Memorial Hospital and Chang Gung University College of Medicine, Kaohsiung, Taiwan; University of São Paulo, Brazil

## Abstract

The levels of circulating microRNAs (miRNAs) in mice with experimental sepsis induced by cecal ligation and puncture (CLP) were determined using whole blood samples obtained from C57BL/6 mice at 4, 8, and 24 h after CLP; miRNA expression analysis was performed in these samples using an miRNA array. Microarray analysis revealed upregulation of 10 miRNA targets (miR-16, miR-17, miR-20a, miR-20b, miR-26a, miR-26b, miR-106a, miR-106b, miR-195, and miR-451). The expression of these miRNA targets in the whole blood, serum, and white blood cells (WBCs) of CLP mice was quantified using quantitative real-time PCR; these values were compared to those in sham-operated C57BL/6 mice, and the results indicated that these miRNA targets were significantly up-regulated in the whole blood and serum but not in the WBCs. In addition, the levels of these 10 miRNA targets in the serum of Tlr2−/−, Tlr4−/−, and NF-κB−/− mice at 8 h after CLP did not decrease significantly., which indicated that the transcription of these miRNAs was not directly mediated by the TLR2/NF-κB or TLR4/NF-κB pathway, and pathways induced by exposure to the gram-positive or gram-negative bacteria. Immunoprecipitation with the Argonaute 2 ribonucleoprotein complex revealed significantly increased expression of the 10 miRNA targets in the serum of mice after CLP, and the levels of 6 (miR-16, miR-17, miR-20a, miR-20b, miR-26a, and miR-26b) of these 10 miRNA targets increased significantly in exosomes isolated using ExoQuick precipitation solution. In this study, we identified circulating miRNAs that were up-regulated after CLP and determined the increase in the levels of these miRNAs, and our results suggest that circulating Ago2 complexes and exosomes may be responsible for the stability of miRNAs in the serum.

## Introduction

Sepsis is a complex clinical condition that results from the inability to of the host to regulate inflammatory responses against infection. Sepsis causes severe adverse outcomes such as shock, organ dysfunction, and death [1]. Despite more than 30 years of extensive study, the pathophysiology of sepsis in humans remains poorly understood, and hospitalization and mortality rates of patients with sepsis have increased substantially [2]. Sepsis accounts for 25% of admissions to the intensive care units (ICUs), and it is associated with a mortality rate of approximately 50% [3]; therefore, there is an urgent need to understand the pathophysiological processes underlying sepsis and identify reliable biomarkers for early diagnosis or therapeutic intervention for this disease.

Various animal models have been used for investigating the pathogenesis of sepsis and for preliminary testing of potential therapeutics. Among these models, cecal ligation and puncture (CLP) in rodents is the most widely used, and it is a realistic model for experimental sepsis, which is currently considered the gold standard in sepsis research [4]–[6]. Ligation of the cecum induces polymicrobial infection and ischemia and provides a source of ischemic tissue. This surgical procedure produces bowel perforation, which results in leakage of feces into the peritoneum and bacterial peritonitis. The combination of ischemic/necrotic tissue and microbial infection distinguishes this multifactorial model from a number of other models of bacterial sepsis [7]. The CLP model reflects the hemodynamic changes and the clinical course of the disease and reproduces the features of the cytokine-and chemokine-mediated immune response observed in human sepsis [8].

Sera and other body fluids contain cell-free DNA, RNA, and circulating nucleic acids, which serve as potential biomarkers for various diseases [9]. Recently, circulating microRNAs (miRNAs) in the serum were found to show specific expression patterns, and these patterns suggested that serum miRNAs contain fingerprints for various diseases; therefore, circulating miRNAs are considered promising markers for the detection of diseases [10]–[12]. miRNAs are approximately 22-nt-long small regulatory RNA molecules that modulate the activity of specific mRNA targets and play important roles in a wide range of physiologic and pathologic processes [13], [14]. Changes in the miRNA expression profiles in tissues have been observed in various diseases, including cancer, cardiovascular and neurological diseases, and several inflammatory and autoimmune diseases. In addition, despite the presence of ubiquitous ribonucleases (RNases), the serum miRNAs levels are remarkably stable and reproducible [15], [16]. Further, biochemical analyses have shown that miRNAs are resistant to RNase activity, extreme pH and temperature, extended storage, and multiple freeze-thaw cycles [17], [18]. At least 2 possible mechanisms–exosome membrane enclosure [19], [20] and conjunction with an RNA-binding protein such as Argonaute 2 (Ago2) [21], [22]–provide a protected and controlled internal microenvironment for the circulating miRNAs and allow them to travel long distances without degradation. A horizontal transfer of the secreted miRNAs from the donor to recipient cells influences gene expression in the recipient cells and function as cell-to-cell communication [23].

Given the possible important role of circulating miRNAs in the regulation of the pathophysiology of various diseases and their potential as disease biomarkers or therapeutic targets, in this study, we aimed to profile the expression of circulating miRNAs in a mouse model of CLP-induced sepsis.

## Materials and Methods

### Animal Experiments

*Tlr2* ^−*/*−^ (B6.129-Tlr2tm1Kir/J), *Tlr4* ^−*/*−^ (C57BL/10ScNJ), and *NF-κB* ^−/−^ (B6.Cg-Nfkb1tm1Bal/J) mice were purchased from Jackson Laboratory (Bar Harbor, ME). C57BL/6 mice were purchased from BioLasco (Taipei, Taiwan). All housing conditions were maintained, and surgical procedures, including analgesia, were performed in an Association for Assessment and Accreditation of Laboratory Animal Care International (AAALAC)-accredited SPF facility according to national and institutional guidelines. Animal protocols were approved by the IACUC of Chang Gung Memorial Hospital (permission number No. 2012091304). Mid-grade sepsis was induced after medium ligation of the cecum according to the established protocol [6]. Briefly, mice were anesthetized with a combination of ketamine and xylazine, and a midline abdominal incision was made. The cecum was mobilized and ligated in the middle of cecum below the ileocecal valve, punctured once using a 21-G needle, and a small stool sample was squeezed out of the cecum to induce polymicrobial peritonitis. The abdominal wall was closed in 2 layers. Sham-operated mice underwent the same procedure, including opening of the peritoneum and exposing the bowel, but without ligation and needle perforation of the cecum. After surgery, the mice were resuscitated by subcutaneous injection of pre-warmed (37°C) normal saline (5 mL per 100 g body weight). To distinguish between circulating miRNA levels in sepsis and bacterial infection, a mouse model with bacterial infection but no progression to sepsis was used. Recombinant-specific gram-negative pathogens *Escherichia coli* (xen14) and gram-positive pathogens *Staphylococcus aureus* (xen29) were purchased from Caliper (Caliper, Princeton, NJ, USA). To induce bacterial infection in the mice, *E. coli* or *S. aureus* suspension (1×10^8^/100 µL of PBS) was injected subcutaneously into the back of the mice by using a Fr. 25 needle. An additional group of animals was inoculated with PBS to serve as a negative control. The mice had *ad libitum* access to food and water both before and after the surgery or administration of bacterial injection. The mice were killed at the indicated time points (4, 8, and 24 h) after the surgery or administration of bacterial injection, and whole blood was drawn.

### Bacterial Cultures

Bacteria were obtained from the ascitic fluid from the abdominal cavity of mice at the indicated times (4, 8, and 24 h) by using sterile cotton swabs. These swabs were transferred to 1 mL of 0.9% normal saline in a sterile tube to obtain a bacterial suspension. Then, the suspension was diluted 100-fold by transferring 0.1 mL to another tube filled with 9.9 mL of 0.9% saline solution. Further, 10 µL of the final diluted suspension was inoculated in 5% sheep blood agar and Eosin-methylene blue agar. The plates were incubated at 37°C for 24–48 h; subsequently, the different bacterial colonies were counted. Cultured bacteria were identified on the basis of colony morphology, Gram staining, and biochemical characteristics by using standard techniques. The growth of ≥1×10^5^ colony-forming units (CFU)/mL was considered clinically significant bacterial colonization. Bacterial contamination was arbitrarily marked as rare (≤1×10^5^ CFU/mL), light (1×10^5^ to 1×10^6^ CFU/mL), and moderate (≥1×10^6^ CFU/mL).

### Collection of Blood Samples and Separation of the Components

Briefly, the whole blood samples (1 mL per mouse) were collected into tubes containing anticoagulant at indicated times. After incubating whole blood at room temperature for 15 min, the samples were centrifuged at 3,000×g for 10 min, white blood cells (WBCs) were slowly removed from the corresponding layers, and the serum was extracted and stored at –80°C before processing for RNA and protein analyses.

### Isolation of Exosomes

Serum samples (250 µL) from sham control animals and mice with CLP for 8 h were thawed on ice and centrifuged at 3000×*g* for 15 min to remove cells and cell debris. Supernatants were transferred to sterile tubes containing 63 µL of ExoQuick precipitation solution (System Biosciences, Mountain View, CA, USA) and mixed. The mixtures were incubated for at least 12 h at 4°C and centrifuged at 1500×*g* for 30 min at 4°C. The supernatants and resuspended exosome pellets were removed with a protein lysis buffer. Further, these vesicles were confirmed as exosomes through western blot analysis using antibodies against 2 commonly used exosomal markers, the tetraspanin molecule CD9 and tumor susceptibility gene 101 (TSG101). Proteins were separated by polyacrylamide gel electrophoresis (PAGE) and electrotransferred to polyvinylidene fluoride (PVDF) membrane (Millipore, Billerica, MA, USA). The membranes were blocked with 5% skim milk in Tween-20/PBS and were probed with rabbit anti-CD9 (1∶1000), rabbit anti-TSG101 (1∶1000), and mouse anti-β-actin (1∶1000) antibodies. Then, the blots were incubated with horseradish peroxidase (HRP)-conjugated secondary antibodies (Santa Cruz, Santa Cruz, CA, USA), and the detected proteins were quantified using a FluorChem 8900 imaging system (Alpha Innotech, San Leandro, CA, USA). The intensity of each signal spot was converted into digital data with autobackground subtraction during spot density analysis using AlphaEaseFC software (Alpha Innotech).

### Immunoprecipitation and Immunoblotting of Ago2

For each sample, 25 µL of Protein A/G plus agarose (Santa Cruz) was washed with PBS buffer (pH 7.4) and incubated with 2 µg of rabbit anti-Ago2 (Abcam, MA, USA) or rabbit normal IgG (Santa Cruz) antibodies for 2 h at 4°C. Beads containing the immobilized anti-Ago2 antibody were then added to 200 µL of serum diluted with 200 µL of PBS and incubated 4 h at 4°C (immunoprecipitation was performed in detergent-free conditions to avoid potential lysis of serum vesicles). Beads were washed 3 times with 1% Nonidet P-40 buffer (1% Nonidet P-40, 50 mM Tris-HCl, pH 7.4, 150 mM NaCl, 2 mM EDTA), and half of each sample was eluted with 2× sodium dodecyl sulfate (SDS) sample buffer and analyzed by SDS/PAGE and immunoblotting with mouse anti-Ago2 antibody (Santa Cruz, CA, USA) to detect Ago2. The remaining half of each sample was eluted with 600 µL of lysis/binding buffer of the mirVana™ miRNA Isolation Kit (Life Technologies, NY, USA) and processed for RNA isolation.

### RNA Isolation and Preparation

Total RNA was extracted from whole blood, serum, WBCs, exosomes, and exosome-depleted supernatants using the mirVana™ miRNA Isolation Kit (Life Technologies, NY, USA). The concentration of RNAs purified was by determining the absorbance at 260 nm using an SSP-3000 Nanodrop spectrophotometer (Infinigen Biotechnology, Inc., City of Industry, CA, USA), the purity of the samples was analyzed using Bioanalyzer 2100 (Agilent Technologies, Santa Clara, CA, USA). Total RNA (10 ng) was reverse transcribed to cDNA by using the TaqMan miRNA Reverse Transcription Kit (Applied Biosystems, Foster City, CA, USA). Target miRNAs were reverse transcribed using sequence-specific stem-loop primers, and cDNA was used for quantitative real-time polymerase chain reaction (qPCR).

### miRNA Microarray Analysis

The Mouse & Rat miRNA OneArray® v3 (Phalanx Biotech Group, Hsinchu, Taiwan), which is based on miRBase Release 17 and which contains 105 experimental control probes, 1111 unique mouse miRNA probes, and 672 rat miRNA probes, was used for the CLP experiments. The Mouse & Rat miRNA OneArray® v4, which is based on miRBase Release 18 and which contains 144 experimental control probes, 1157 unique mouse miRNA probes, and 680 rat miRNA probes was used for the subcutaneous bacterial injection experiment. Mouse genome-wide miRNA microarray analysis and statistical analysis was performed by Phalanx Biotech. Briefly, fluorescent targets were prepared from 2.5 µg of total RNA by using the miRNA ULS™ Labeling Kit (Kreatech Diagnostics, Amsterdam, Netherlands). Labeled miRNA targets enriched using NanoSep 100K (Pall Corporation, Port Washington, NY, USA) were hybridized to the Mouse & Rat miRNA OneArray® v3 in the Phalanx hybridization buffer by using the OneArray® Hybridization Chamber. After overnight hybridization at 37°C, non-specifically bound targets were removed through 3 washing steps (Wash I, 37°C, 5 min; Wash II, 37°C, 5 min and 25°C, 5 min; and Wash III, rinse 20 times). The slides were dried by centrifugation and were scanned using an Axon 4000B scanner (Molecular Devices, Sunnyvale, CA, USA). The signal intensities of Cy5-fluorescence in each spot were analyzed using GenePix 4.1 software (Molecular Devices, CA, USA) and processed using the R language (http://www.r-project.org/) with the following 2 packages: limma (http://www.bioconductor.org/packages/release/bioc/html/limma.html) and genefilter (http://www.bioconductor.org/packages/release/bioc/html/genefilter.html). We filtered out spots that were flagged <0, and spots that passed this criteria were normalized using 75% media-scaling normalization method. Normalized spot intensities were converted into gene expression log_2_ ratios for the control and treatment groups. Spots with log_2_ ratios ≤–1 or ≥1 and *P*-value <0.05 were selected for further analysis. The differentially expressed miRNAs were subjected to hierarchical cluster analysis, and average linkage and Pearson correlation were as measures of similarity. The miRNA array and the microarray data have been deposited in Gene Expression Omnibus (accession numbers: GSE47094 & GSE49189, respectively).

### Quantification of miRNA Expression

miRNA expression was quantified using qPCR in an Applied Biosystems 7500 Real*-*Time PCR System (Life Technologies) to confirm the upregulation of expression of miRNA targets that were detected in the miRNA array from whole blood collected at the indicated times after CLP (4, 8, and 24 h). The expression level of each miRNA in whole blood or WBC samples was represented relative to the expression of U6 small nuclear RNA (*U6 snRNA*), which was used as an internal control. For the serum, exosome, and exosome-depleted supernatants, 25 fmol of single-stranded cel-miR-39 synthesized by Invitrogen (Carlsbad, CA, USA) was added to 400 µL of serum as an internal control for expression of each miRNA. We evaluated the expression levels by calculating the relative expression values in 6 samples and comparing these values to that from the control samples; induction was expressed as fold-change in miRNA expression relative to that in the control. Analysis of variance (ANOVA) was used for intergroup comparisons, and an appropriate post-hoc test was used to compensate for multiple comparisons (SigmaStat, Jandel,CA, USA). *P* values <0.05 were considered significant.

## Results

### Bacterial Yield from Culture of Ascitic Fluid

**Figure 1:**
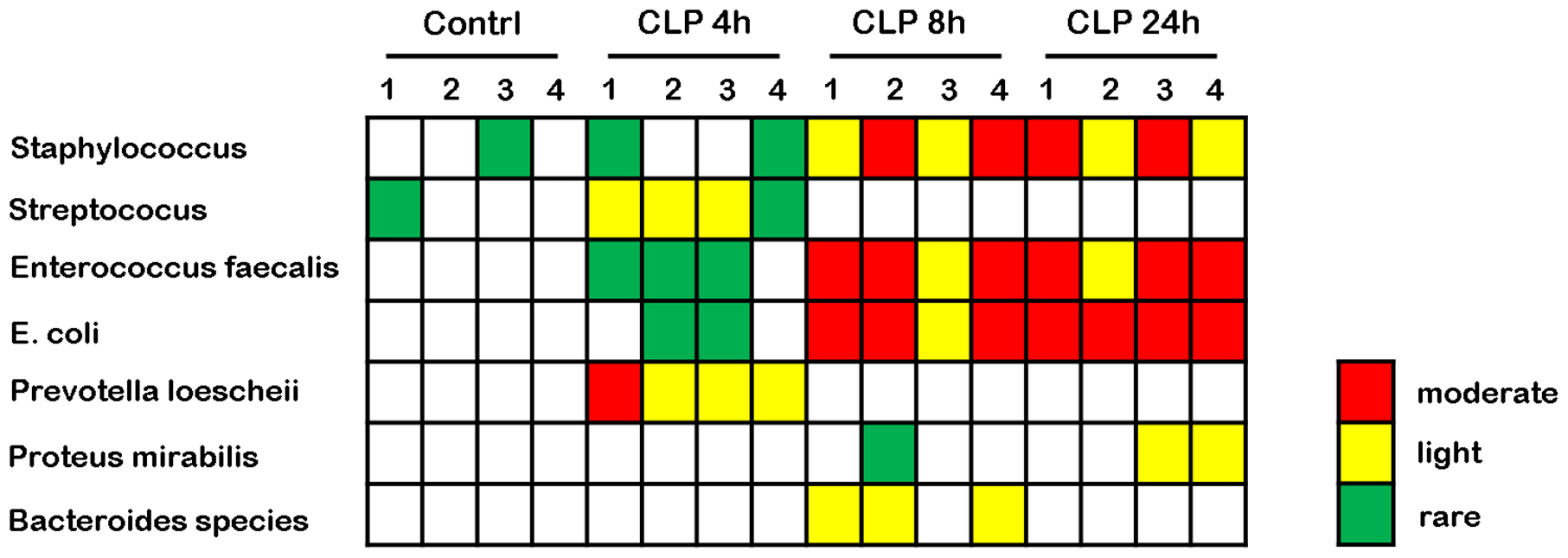
Bacterial counts in ascites of mice at 4, 8, and 24 h following cecal ligation and puncture. Bacterial contamination was arbitrarily marked as rare (≤1×10^5^ CFU/mL), light (1×10^5^ to 1×10^6^ CFU/mL), or moderate (≥1×10^6^ CFU/mL) and are indicated in green, yellow, and red, respectively.

Cultures of ascitic fluid of mice subjected to CLP showed mixed infection with gram-positive, gram-negative, and anaerobic bacteria (Fig. 1). When bacterial contaminations was assessed as light or moderate, microorganisms isolated from the ascitic fluid of mice at 4 h after CLP were identified as *Streptococcus* species (3/4) and *Prevotella loescheii* (4/4), whereas those in the ascitic fluid of mice at 8 h after CLP were identified as *Staphylococcus* species (4/4), *Enterococcus faecalis* (4/4), *E. coli* (4/4), and *Bacteroides* species (3/4). At 24 h, the organisms isolated from the ascitic fluid cultures were *Staphylococcus* species (4/4), *E. faecalis* (4/4), *E. coli* (4/4), and *Proteus mirabilis* (2/4). The ascitic fluid cultures continued to show mixed infection with bacterial contamination increasing with time.

### Up-regulated miRNA Targets in Microarray Analysis

**Figure 2:**
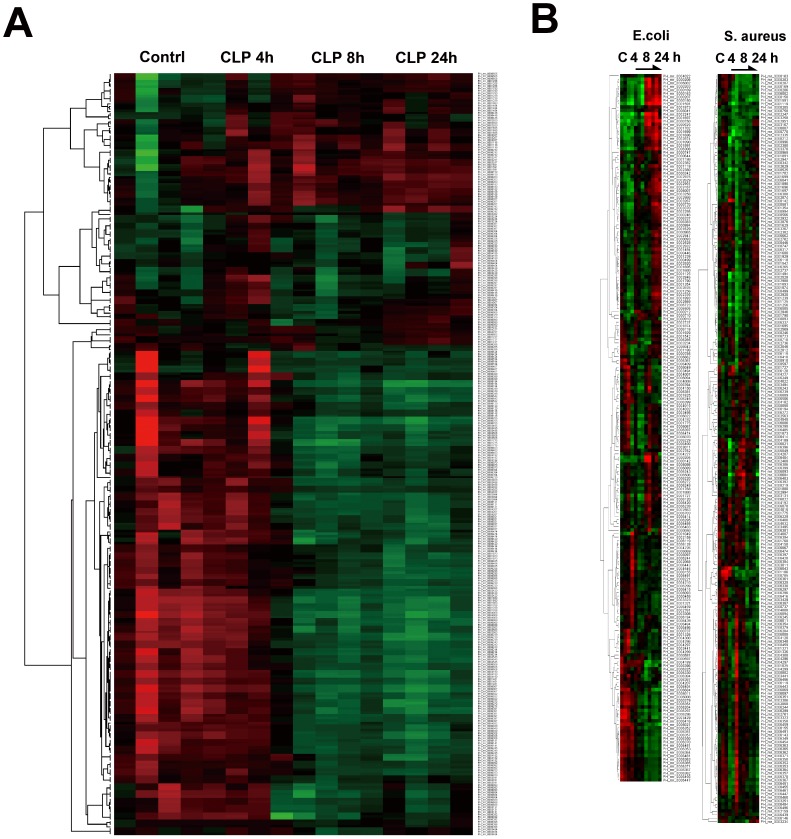
Hierarchical cluster analysis. Clustering of differentially expressed circulating microRNAs in the whole blood of C567BL/6 mice at 4, 8, and 24 h after cecal ligation and puncture (A) and subcutaneous bacteria injection with 1×10^8^ *E. coli.* or *S. aureus* (B).

**Figure 3:**
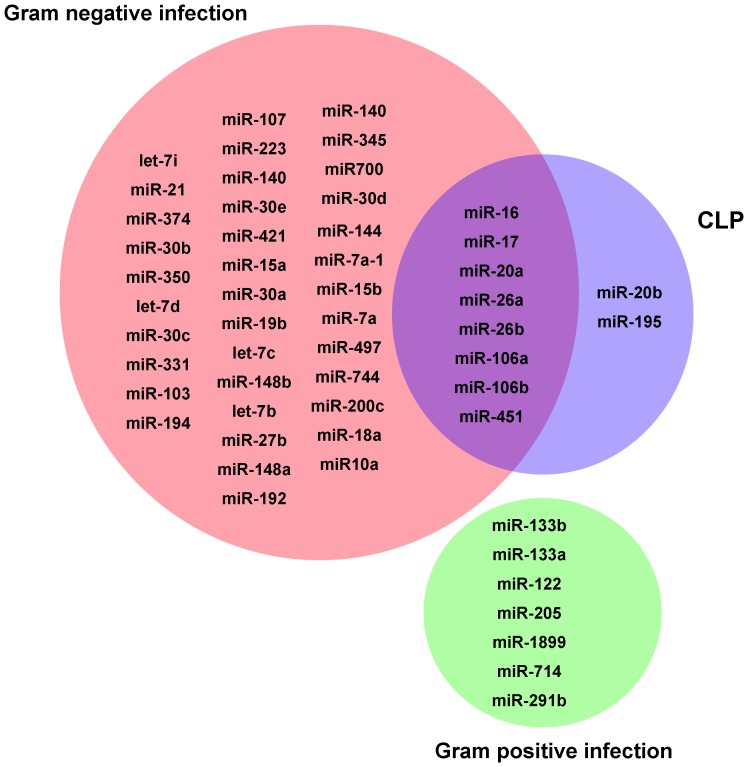
Up-regulated microRNA targets. Venn diagram showing the common and specific up-regulated miRNAs in the cecal ligation and puncture experiment without and after subcutaneous injection of gram-negative or gram-positive bacteria.

Compared to the control group, the experimental group showed more than 2-fold difference in the expression of miRNAs in the whole blood samples at 4, 8, or 24 h (*P*<0.05; n = 4 for each subgroup). The hierarchical cluster analysis of all miRNAs differentially expressed in whole blood after CLP and subcutaneous bacterial injection is shown in Fig. 2A and 2B, respectively. In both experiments, unsupervised hierarchical clustering was used to separate the samples from experimental subjects or control subjects into different groups. The miRNA targets that were significantly up-regulated in the CLP experiment, as shown by the microarray experiments, are shown in Table 1. The expressions of 2 (miR-16 and miR-17), 6 (miR-20a, miR-16, miR-17, miR-451, miR-106a, and miR-106b), and 7 miRNAs (miR-26b, miR-20b, miR-17, miR-20a, miR-106a, miR-26a, and miR-195) increased significantly in the whole blood of mice at 4, 8, and 24 h after CLP, respectively. Therefore, 10 miRNAs were deemed as the up-regulated miRNA targets in the whole blood after CLP, and these 10 were subjected to further validation using qPCR. In addition, the miRNA targets that were significantly up-regulated in the microarray analysis in mice subcutaneously injected with gram-negative *E. coli* and gram-positive *S. aureus* are shown in Table 2 and Table 3, respectively. In the microarray analyses, no up-regulated miRNAs were detected at 4 h after subcutaneous injection. At 8 h after exposure injection, the level of 6 miRNAs (miR-451, miR-122, miR-350, miR-301a, miR-126, and miR-331) in the whole blood increased significantly. At 24 h after injection, the levels of 45 miRNAs increased in the whole blood (Table 2). In addition, after subcutaneous injection of *S. aureus*, no up-regulated miRNAs were detected in the whole blood at 4 h and 8 h. At 24 h after *S. aureus* injection, the levels of 7 miRNAs (miR-133b, miR-133a, miR-122, miR-205, miR-1899, miR-714, and miR-291b) increased significantly (Table 3). These results show that 8 miRNAs (miR-16, miR-17, miR-20a, miR-26a, miR-26b, miR-106a, miR-106b, and miR-451) were up-regulated after both CLP and subcutaneous injection of *E. coli*. Two miRNAs (miR-20b and miR-195) were only significantly expressed in the CLP model, but were not expressed after injection of *E. coli*. Furthermore, in the mice injected with *S. aureus*, no up-regulated miRNA targets common to both CLP experiment group and the *S. aureus-*infected group. The common and specific up-regulated miRNAs in the CLP and bacterial infection groups are shown Venn diagram in Figure 3.

**Table 1:** microRNA targets up-regulated more than 2-fold in whole blood of mice at 4, 8 and 24 h after cecal ligation and puncture (*P*-value <0.05).

CLP 4 h	Fold (log_2_)	p-value	CLP 8 h	Fold (log_2_)	p-value	CLP 24 h	Fold (log_2_)	p-value
mmu-miR-16	1.33	0.021	mmu-miR-20a	1.10	0.033	mmu-miR-26b	1.17	0.028
mmu-miR-17	1.68	0.041	mmu-miR-16	1.13	0.033	mmu-miR-20b	1.35	0.003
			mmu-miR-17	1.25	0.025	mmu-miR-17	1.38	0.015
			mmu-miR-451	1.35	0.034	mmu-miR-20a	1.42	0.006
			mmu-miR-106a	1.39	0.038	mmu-miR-106a	1.54	0.036
			mmu-miR-106b	1.73	0.034	mmu-miR-26a	1.56	0.049
						mmu-miR-195	2.45	0.017

Up-regulated microRNA targets (more than 2-fold difference with control; *P*-value <0.05) in samples from whole blood of experimental mice at 4, 8, and 24 h after cecal ligation and puncture.

**Table 2:** microRNA targets up-regulated more than 2-fold in whole blood of mice at 4, 8 and 24 h after subcutaneous injection of 1×10^8^ cells of *Escherichia coli* (*P*-value <0.05).

4 h	Fold(log_2_)	p-value	8 h	Fold (log_2_)	p-value	24 h	Fold(log_2_)	p-value
Nil			mmu-miR-451	1.521	0.014	mmu-miR-16	3.703	0.001
			mmu-miR-122	1.117	0.003	mmu-miR-26a	3.532	0.005
			mmu-miR-350	1.105	0.014	mmu-miR-20a	2.832	0.011
			mmu-miR-301a	1.038	0.050	mmu-let-7i	2.759	0.003
			mmu-miR-126	1.028	0.046	mmu-miR-21	2.692	0.007
			mmu-miR-331	1.017	0.031	mmu-miR-374	2.666	0.021
						mmu-miR-17	2.471	0.002
						mmu-miR-30b	2.425	0.007
						mmu-miR-26b	2.312	0.019
						mmu-miR-350	2.126	0.008
						mmu-miR-106a	2.105	0.027
						mmu-let-7d	2.096	0.004
						mmu-miR-106b	2.093	0.009
						mmu-miR-30c	1.991	0.002
						mmu-let-7a	1.933	0.033
						mmu-miR-331	1.931	0.005
						mmu-miR-103	1.872	0.007
						mmu-miR-194	1.842	0.004
						mmu-miR-107	1.834	0.010
						mmu-miR-223	1.770	0.000
						mmu-miR-140	1.738	0.002
						mmu-miR-30e	1.705	0.021
						mmu-miR-421	1.636	0.001
						mmu-miR-15a	1.627	0.005
						mmu-miR-30a	1.569	0.003
						mmu-miR-19b	1.530	0.028
						mmu-let-7c	1.517	0.017
						mmu-miR-148b	1.455	0.014
						mmu-let-7b	1.388	0.008
						mmu-miR-27b	1.274	0.002
						mmu-miR-148a	1.270	0.002
						mmu-miR-192	1.231	0.001
						mmu-miR-140	1.196	0.008
						mmu-miR-345	1.193	0.020
						mmu-miR-700	1.185	0.025
						mmu-miR-30d	1.175	0.026
						mmu-miR-144	1.147	0.009
						mmu-miR-7a-1	1.131	0.004
						mmu-miR-15b	1.063	0.006
						mmu-miR-451	1.063	0.028
						mmu-miR-7a	1.046	0.000
						mmu-miR-497	1.044	0.002
						mmu-miR-744	1.011	0.000
						mmu-miR-200c	1.006	0.001
						mmu-miR-18a	1.004	0.032
						mmu-miR-10a	1.004	0.008

**Table 3:** microRNA targets up-regulated more than 2-fold in whole blood of mice at 4, 8 and 24 h after subcutaneous injection of 1×10^8^ cells of *Staphylococcus aureus* (*P*-value <0.05).

4 h	Fold (log_2_)	p-value	8 h	Fold (log_2_)	p-value	24 h	Fold (log_2_)	p-value
Nil			Nil			mmu-miR-133b	1.632	0.009
						mmu-miR-133a	1.333	0.011
						mmu-miR-122	1.278	0.049
						mmu-miR-205	1.185	0.029
						mmu-miR-1899	1.105	0.008
						mmu-miR-714	1.088	0.007
						mmu-miR-291b	1.040	0.002

### Expression Profiles of miRNAs

**Figure 4:**
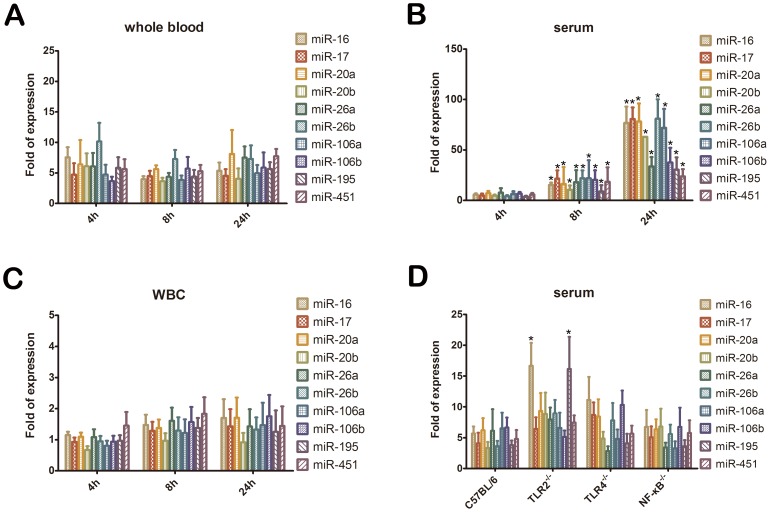
Quantification of up-regulated microRNA targets detected using an miRNA array by quantitative real-time polymerase chain reaction at 4, 8, and 24 h after cecal ligation and puncture. (A) whole blood; (B) sera; (C) WBCs; (D) sera of Tlr2^−/−^, Tlr4^−/−^, and NF-κB^−/−^, and wild-type mice 8 h after CLP. Bars represent means ± SEM of 6 experiments; *, *P*<0.05 vs. control.

The expression of the selected miRNA targets in the whole blood, serum, and WBCs was quantified by qPCR to verify upregulation that was detected in the whole blood samples of CLP-treated mice using miRNA microarray. Significantly increased expression (5- to 10-fold) was observed for all 10 up-regulated miRNA targets throughout the experiment (Fig. 4A). In the serum, all 10 up-regulated miRNA targets showed time-dependent increase after CLP. Significant expression of these 10 miRNA targets was detected at 8 and 24 h after CLP, and 6 targets (miR-16, miR-17, miR-20a, miR-20b, miR-26b, miR-106a) showed remarkable upregulation of up to 50- and even 100-fold at 24 h (Fig. 4B). In contrast, in WBCs, no significant increase of the 10 miRNA targets was observed after CLP (Fig. 4C).

The level of these 10 up-regulated miRNA targets did not decrease significantly in the serum of *Tlr2* ^−*/*−^, *Tlr4* ^−*/*−^, and *NF-κB* ^−/−^ mice at 8 h after CLP, which implied that the transcription of these 10 miRNAs was not directly mediated by the TLR2/NF-κB or TLR4/NF-κB pathways, which are induced by exposure to the gram-positive or gram-negative bacteria (Fig. 4D). Moreover, compared to CLP-treated wild-type mice, *Tlr2* ^−*/*−^ mice with CLP showed significantly increased serum miR-16 and miR-195 levels.

### miRNA Levels in Exosomes

**Figure 5:**
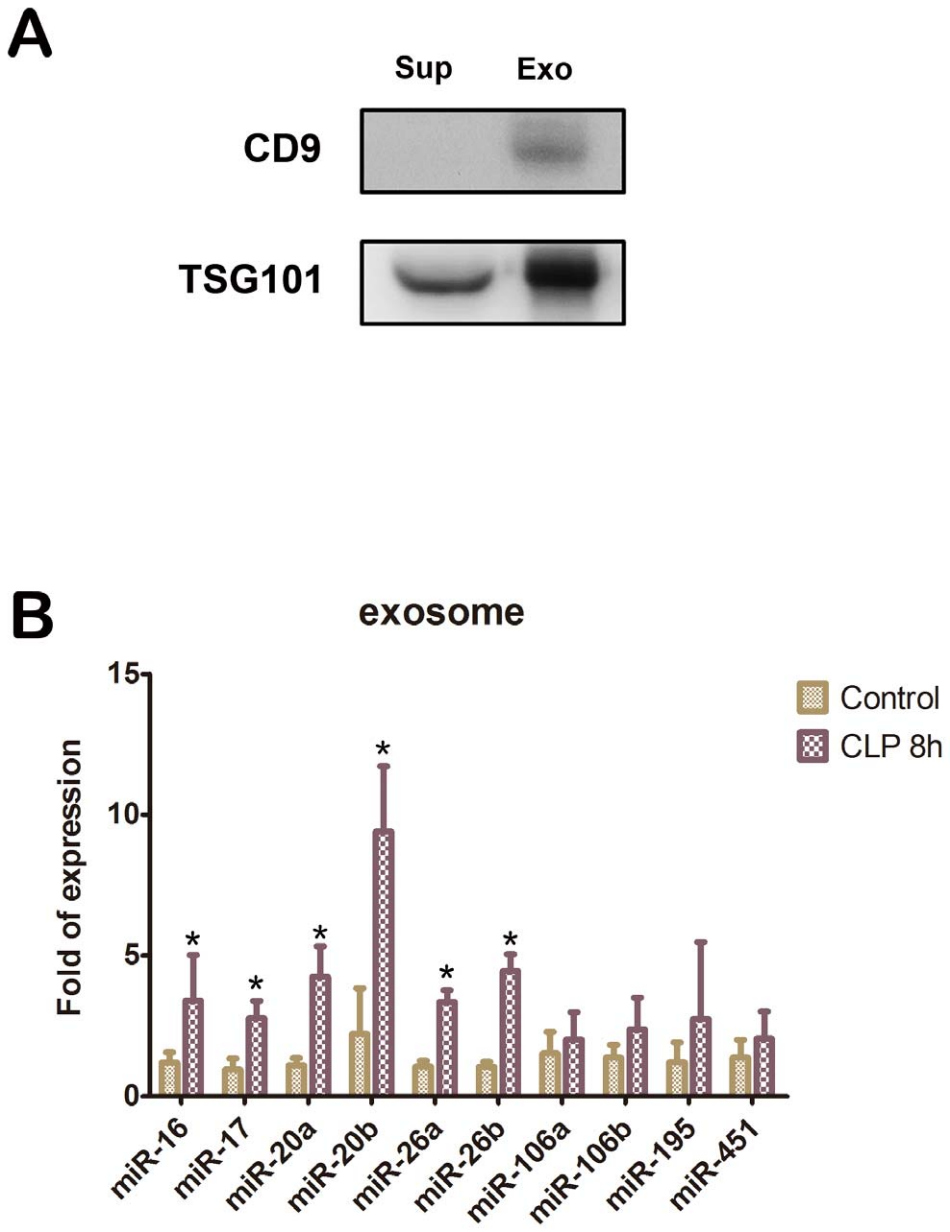
Expression of the up-regulated microRNA targets in the exosomes. (A) Western blot analysis of the ExoQuick-captured pellets with antibodies against exosomal markers CD9 and TSG101. Sup: supernatant; Exo: exosomes from the ExoQuick-captured pellet. (B) Up-regulated microRNA targets quantified by quantitative real-time polymerase chain reaction in exosomes of C57BL/6 mice at 8 h after cecal ligation and puncture. Bars represent means ± SEM of 6 experiments; *, *P*<0.05 vs. control.

Presence of exosomes in the pellets obtained after ultracentrifugation was confirmed by western blot analysis using antibodies against 2 exosomal membrane markers, the tetraspanin molecule CD9 and TSG101. Western blot showed strong staining of the ExoQuick-captured pellet with the exosomal membrane markers anti-CD9 and anti-TSG101 (Fig. 5A). The exosomes showed marked expression of 6 (miR-16, miR-17, miR-20a, miR-20b, miR-26a, and miR-26b) of the 10 miRNA targets at 8 h after CLP; the expression of the remaining 4 miRNA targets (miR-106a, miR-106b, miR-195, and miR-451) increased; however, the increase was not significant (Fig. 5B).

### miRNA Expression in Ago2 Complexes

**Figure 6:**
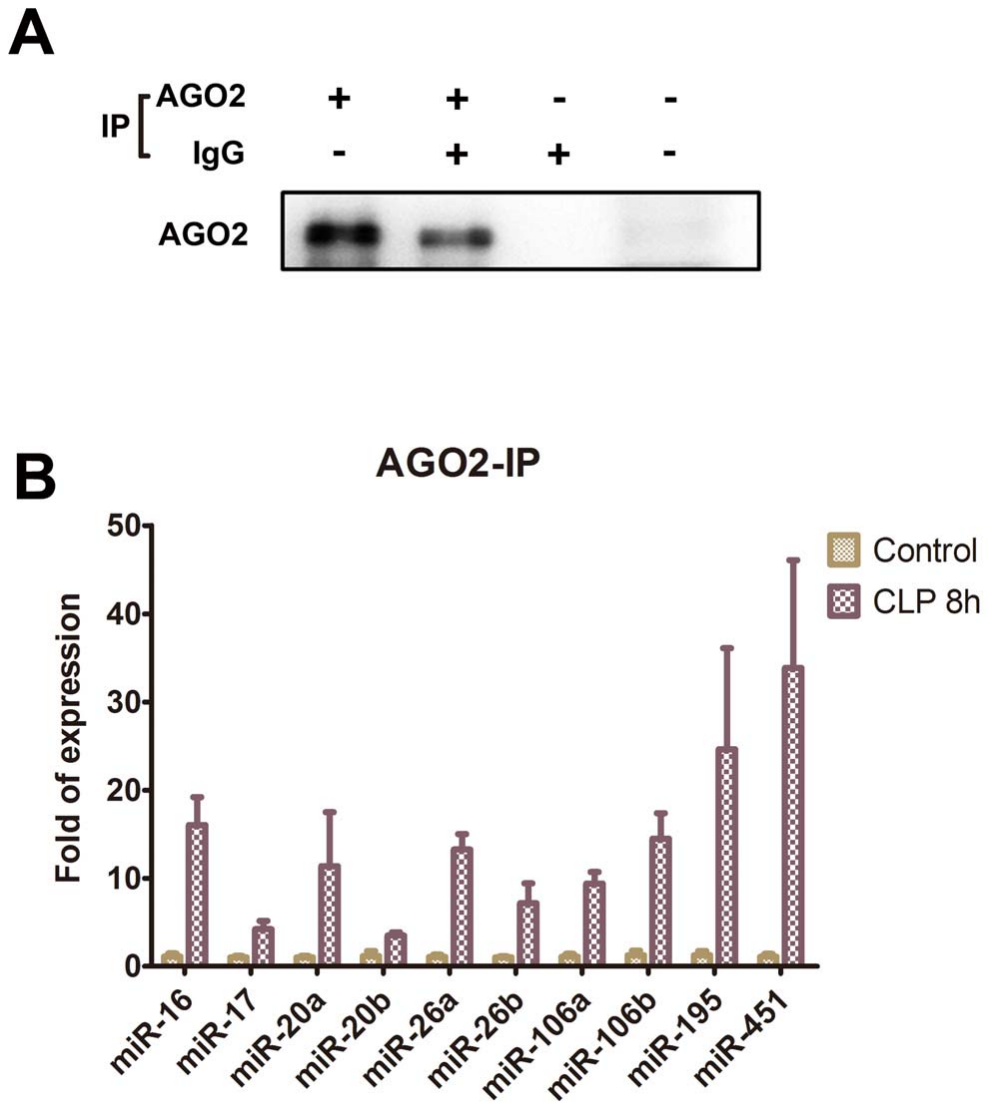
Expression of the up-regulated microRNA targets in the Ago2 ribonucleoprotein complex. (A) Immunoprecipitation and immunoblotting with antibodies against Ago2 with or without IgG competition; (B) Up-regulation of microRNA targets associated with Ago2 complexes in the serum of C57BL/6 mice at 8 h after cecal ligation and puncture quantified by quantitative real-time polymerase chain reaction. Bars represent means± SEM of 6 experiments; *, *P*<0.05 vs. control.

Presence of Ago2 proteins in the serum was determined by immunoprecipitation followed by immunoblotting with antibodies against Ago2 protein (Fig. 6A). Immunoprecipitation against Ago2 in the presence of IgG decreased the Ago2 immunoprecipitates. In addition, the absence of Ago2 in the negative control IgG immunoprecipitates showed that Ago2 was specifically precipitated from the serum. Without immunoprecipitation, Ago2 levels in serum were below the limit of detection by immunoblotting. To determine whether circulating miRNAs are associated with Ago2, the Ago2 immunoprecipitates were assayed for the 10 up-regulated miRNA targets, and the results revealed that the expression of all the 10 miRNA targets was significantly up-regulated in the serum at 8 h following CLP (Fig. 6B). Among these miRNAs, the levels of miR-195 and miR-451 detected in the Ago2 immunoprecipitates were more than 20-fold that in the controls and the levels of miR-16, miR-20a, miR-26a, and miR-106b were more than 10-fold that in the control.

## Discussion

In this study, we demonstrated that experimental sepsis induced by CLP caused time-dependent upregulation of the circulating miRNAs miR-16, miR-17, miR-20a, miR-20b, miR-26a, miR-26b, miR-106a, miR-106b, miR-195, and miR-451. The up-regulated miRNAs were detected in the serum but not in the blood cellular components such as WBCs. In addition, all the up-regulated miRNAs were present in a non–membrane-bound form consistent with Ago2 ribonucleoprotein complexes, and some these miRNAs were associated with exosomes in the serum. Recent studies suggested that 90% of the serum miRNAs are bound to protein complexes such as Ago2, RNA-binding protein nucleophosmin 1 (NPM1), and HDL [24]. Ago2 binds miRNAs with high affinity (Kd = 10–80 nM for tagged mammalian Ago2 binding to mature miRNAs) [25], [26], and association of Ago2 with serum miRNAs can therefore greatly influence miRNA stability in the serum. Because the efficiency of Ago2 immunoprecipitation and exosome capture with ExoQuick is likely less than 100%, the estimated levels may not account for the Ago2- and exosome-associated miRNAs in the serum. In fact, Arroyo et al. [21] estimated that efficiency of Ago2 immunoprecipitation from cell lysates was, at most, 60% and demonstrated that some amounts of the assayed miRNA remained in the supernatant following immunoprecipitation with Ago2, although miRNAs were consistently detected in complexes with Ago2. Therefore, our results strongly support the observation that circulating Ago2/miRNA complexes serve as carriers of miRNAs in serum.

Although increases in circulating miRNAs originating from LPS-stimulated monocytes and monocyte-derived dendritic cells during sepsis have been reported [27], [28], the origins of circulating miRNAs after CLP have not been identified. In this study, we found that none of the up-regulated miRNAs was expressed in the WBCs. Previously, we had demonstrated a dose- and time-dependent up-regulation of 8 (let-7d, miR-15b, miR-16, miR-25, miR-92a, miR-103, miR-107, and miR-451) and 4 (miR-451, miR-668, miR-1902, and miR-1904) circulating miRNA targets in mice following injection of LPS [11] and LTA [12], respectively. However, in the CLP-based model of sepsis, only miR-16 was up-regulated. In addition, the level of none of the 10 miRNA targets was significantly lower in the sera of CLP-treated *Tlr2* ^−*/*−^, *Tlr4* ^−*/*−^, and *NF-κB* ^−/−^ mice compared to that in wild-type mice, indicating that the transcription of these miRNAs is not directly mediated by the TLR2/NF-κB or TLR4/NF-κB pathways, which are triggered by direct exposure to gram-positive or gram-negative bacteria. In this study, the mice with CLP experienced bacterial infection first and then septicemia, therefore, the results clearly showed that 8 miRNA targets (miR-16, miR-17, miR-20a, miR-26a, miR-26b, miR-106a, miR-106b, and miR-451) were up-regulated in both the CLP alone group and the *E. coli* infection group. Furthermore, up-regulated miRNA target was common to both the CLP group and the *S. aureus* infection group. This result also highlights the dominant role of gram-negative bacteria in the expression of circulating miRNAs during the progress of bacterial infection to sepsis in the CLP model. In this study, 2 miRNAs (miR-20b and miR-195) were significantly expressed in the CLP model, but not after exposure to *E. coli*. The expression of these 2 miRNAs might be related to the sepsis-related pathophysiological responses, but not to exposure to gram-negative bacteria. Pathophysiological responses related to sepsis such as inflammation, shock, or even ileus in the CLP model might also contribute to the upregulation in the expression of circulating miRNAs; however, these hypotheses require further investigation. Further investigation of different downstream adaptors such as Myd88, Trif, Rip2, and ASC using knockout mice might be beneficial for elucidating the function of innate immunity in the upregulation of these circulating miRNAs.

Circulating miR-15a and miR-16 [29], [30], miR-150 [31], miR-146a, and miR-223 [32] have been identified as potential biomarkers of sepsis. Serum levels of both miR-15a and miR-16 were significantly higher in patients with sepsis that in healthy individuals [29] or cirrhotic patients [30], and the levels of these miRNAs were significantly elevated in patients with systemic inflammatory response syndrome (SIRS) [29]. Expression of circulating miR-16 was up-regulated ∼5-fold following LPS treatment [11] and ∼70-fold at 24 h following CLP treatment, as demonstrated in this study. On the other hand, the levels of some of the identified miRNAs (miR-150, miR-146a, and miR-223) were significantly reduced in sepsis patients. miR-150 levels were shown to be significantly down-regulated in sepsis, which correlated with disease severity [31]. Similarly, miR-150 concentrations in the serum of ICU patients upon admission closely correlated with their immediate and long-term survival rates, and low miR-150 levels was indicative of unfavorable prognosis [33]. In addition, the miR-146a and miR-223 expression levels were significantly lower in the sera of sepsis patients than in the sera of SIRS patients and normal controls [32]. However, considering transcriptional regulation of miRNA expression in response to physiological or pathological changes, the up-regulated miRNA targets seem better suited for use as biomarkers.

In this study, synthetic *Caenorhabditis elegans* miRNAs were used as endogenous controls for qPCR. Although in some previous studies miR-16 and miR-142 showed relatively stable expression in the serum, owing to which these were used as endogenous controls [34], [35], studies have also shown that miR-16 and miR-451 expressed in red blood cells were the major source of variation in the estimated levels of circulating miR-16 and miR-451 [36]. In addition, miR-16 expression is up-regulated following LPS treatment [11], and it was shown to be up-regulated following CLP in this study, which precludes the use of miR-16 as internal control.

Currently, little is known about the biological roles of these molecules [37]. Among the circulating miRNAs that are up-regulated following CLP, miR-16 has been suggested to regulate pro-apoptotic pathways in circulating lymphocytes of patients with bacterial infections [30]. miR-17 and miR-20a belong to a group of commonly overexpressed miRNAs, the miR-17∼92 cluster, which is located on mouse chromosome 14 (13 in humans) and comprises 7 mature miRNAs (miR-17-5p and, miR-18a, miR-19a and b, miR-20a, and miR-92a). This cluster is directly transactivated by c-Myc, a transcription factor initially linked to tumorigenesis [38], which has made the miR-17∼92 cluster the focus of attention in higher vertebrate research. However, this cluster is also involved in the regulation of hematopoiesis and immune functions [39], and it appears as a global regulator of the apoptosis signal-regulating kinase 1 (ASK1) signalosome, a central effector in the inflammatory pathways [40]. Numerous components of the “extended” ASK1 signalosome are potentially targeted by miRNAs encoded by the miR-17∼92 cluster [40]. By targeting Ask1 mRNA, miR-20 effectively controls the production of inflammatory cytokines by fibroblast-like synoviocytes in response to stimulation by a TLR4 ligand LPS [41].

The circulating miRNA targets that were up-regulated following CLP belong not only to the miR-17∼92 cluster but also to its evolutionary paralogs, miR-106a∼363 (miR-106a, miR-18b, miR-20b, miR-19b-2, miR-92a-2, and miR-363) and miR-106b∼93 (miR-106b, miR-93, miR-25). miR-17, miR-20a, and miR-106a all specifically bind to the same seed sequence within the 3′-untranslated region (UTR) of signal-regulatory protein α (SIRPα), an essential signaling molecule that modulates leukocyte-mediated inflammatory responses and are inversely correlated with SIRPα expression in various cells [42]. Both in vitro and in vivo assays demonstrate that miR-17, miR-20a, and miR-106a regulate macrophage infiltration, phagocytosis, and proinflammatory cytokine secretion by targeting SIRPα [42]. Gain- and loss-of-function studies showed that miR-26a/b directly suppressed the expression of CDK6 and cyclin E1 and blocked G1/S-phase progression [43]. miR-26a may directly regulate the production of IFN-β in human cells [44], which is one of the first steps in the innate immune response to viral infections. Respiratory syncytial virus infection of human alveolar epithelial (A549) cells induced the expression of host miRNAs, including miR-26b, which affected the antiviral response of the host [45]. miR-195 has a complementary site in the 3′-UTR of a membrane water channel protein Aquaporin 8 (AQP8), thereby modulating AQP8 expression [46]. AQP8 expression decreased in the colon of patients with ulcerative colitis [46], and this reduction appears to correlate with disease activation in patients with diarrhea-predominant irritable bowel syndrome [47]. miR-195 may also exert its tumor suppressive effects by decreasing the expression of multiple NF-κB downstream effectors via direct targeting of IKKα and TAB3 [48]. Finally, miR-451 has been reported as a promising biomarker for *Actinobacillus pleuropneumoniae* infection in lung tissues [49].

Although some functions of the circulating miRNAs up-regulated by CLP have been discussed above, further studies are required to clarify the pathophysiological role of each circulating miRNA in sepsis before considering these miRNAs as biomarkers for sepsis.

## References

[1] Warren HS (1997) Strategies for the treatment of sepsis. New England Journal of Medicine 336: 952–953. 9070479 10.1056/NEJM199703273361311

[2] Dombrovskiy VY, Martin AA, Sunderram J, Paz HL (2007) Rapid increase in hospitalization and mortality rates for severe sepsis in the United States: a trend analysis from 1993 to 2003. Crit Care Med 35: 1244–1250. 17414736 10.1097/01.CCM.0000261890.41311.E9

[3] Engel C, Brunkhorst FM, Bone HG, Brunkhorst R, Gerlach H, et al (2007) Epidemiology of sepsis in Germany: results from a national prospective multicenter study. Intensive Care Med 33: 606–618. 17323051 10.1007/s00134-006-0517-7

[4] Rittirsch D, Hoesel LM, Ward PA (2007) The disconnect between animal models of sepsis and human sepsis. J Leukoc Biol 81: 137–143. 17020929 10.1189/jlb.0806542

[5] Buras JA, Holzmann B, Sitkovsky M (2005) Animal models of sepsis: setting the stage. Nat Rev Drug Discov 4: 854–865. 16224456 10.1038/nrd1854

[6] Rittirsch D, Huber-Lang MS, Flierl MA, Ward PA (2009) Immunodesign of experimental sepsis by cecal ligation and puncture. Nat Protoc 4: 31–36. 19131954 10.1038/nprot.2008.214 PMC2754226

[7] Cuenca AG, Delano MJ, Kelly-Scumpia KM, Moldawer LL, Efron PA (2010) Cecal ligation and puncture. Curr Protoc Immunol 19: 13. 21053304 10.1002/0471142735.im1913s91 PMC3058382

[8] Remick DG, Newcomb DE, Bolgos GL, Call DR (2000) Comparison of the mortality and inflammatory response of two models of sepsis: lipopolysaccharide vs. cecal ligation and puncture. Shock 13: 110–116. 10670840 10.1097/00024382-200013020-00004

[9] Swarup V, Rajeswari MR (2007) Circulating (cell-free) nucleic acids–a promising, non-invasive tool for early detection of several human diseases. FEBS Lett 581: 795–799. 17289032 10.1016/j.febslet.2007.01.051

[10] Vlassov AV, Magdaleno S, Setterquist R, Conrad R (2012) Exosomes: Current knowledge of their composition, biological functions, and diagnostic and therapeutic potentials. Biochim Biophys Acta 7: 940–948. 10.1016/j.bbagen.2012.03.017 22503788

[11] Hsieh CH, Rau CS, Jeng JC, Chen YC, Lu TH, et al (2012) Whole blood-derived microRNA signatures in mice exposed to lipopolysaccharides. J Biomed Sci 19: 1423–0127. 10.1186/1423-0127-19-69 PMC3419134 22849760

[12] Hsieh CH, Yang JC, Jeng JC, Chen YC, Lu TH, et al (2013) Circulating microRNA signatures in mice exposed to lipoteichoic acid. J Biomed Sci 20: 1423–0127. 10.1186/1423-0127-20-2 PMC3568731 23286671

[13] Kloosterman WP, Plasterk RH (2006) The diverse functions of microRNAs in animal development and disease. Dev Cell 11: 441–450. 17011485 10.1016/j.devcel.2006.09.009

[14] Stefani G, Slack FJ (2008) Small non-coding RNAs in animal development. Nat Rev Mol Cell Biol 9: 219–230. 18270516 10.1038/nrm2347

[15] Mitchell PS, Parkin RK, Kroh EM, Fritz BR, Wyman SK, et al (2008) Circulating microRNAs as stable blood-based markers for cancer detection. Proc Natl Acad Sci U S A 105: 10513–10518. 18663219 10.1073/pnas.0804549105 PMC2492472

[16] Kosaka N, Iguchi H, Yoshioka Y, Takeshita F, Matsuki Y, et al (2010) Secretory mechanisms and intercellular transfer of microRNAs in living cells. J Biol Chem 285: 17442–17452. 20353945 10.1074/jbc.M110.107821 PMC2878508

[17] Chen X, Ba Y, Ma L, Cai X, Yin Y, et al (2008) Characterization of microRNAs in serum: a novel class of biomarkers for diagnosis of cancer and other diseases. Cell Research 18: 997–1006. 18766170 10.1038/cr.2008.282

[18] Mitchell PS, Parkin RK, Kroh EM, Fritz BR, Wyman SK, et al (2008) Circulating microRNAs as stable blood-based markers for cancer detection. Proceedings of the National Academy of Sciences of the United States of America 105: 10513–10518. 18663219 10.1073/pnas.0804549105 PMC2492472

[19] Skog J, Wurdinger T, van Rijn S, Meijer DH, Gainche L, et al (2008) Glioblastoma microvesicles transport RNA and proteins that promote tumour growth and provide diagnostic biomarkers. Nat Cell Biol 10: 1470–1476. 19011622 10.1038/ncb1800 PMC3423894

[20] Valadi H, Ekstrom K, Bossios A, Sjostrand M, Lee JJ, et al (2007) Exosome-mediated transfer of mRNAs and microRNAs is a novel mechanism of genetic exchange between cells. Nat Cell Biol 9: 654–659. 17486113 10.1038/ncb1596

[21] Arroyo JD, Chevillet JR, Kroh EM, Ruf IK, Pritchard CC, et al (2011) Argonaute2 complexes carry a population of circulating microRNAs independent of vesicles in human plasma. Proc Natl Acad Sci U S A 108: 5003–5008. 21383194 10.1073/pnas.1019055108 PMC3064324

[22] Wang K, Zhang S, Weber J, Baxter D, Galas DJ (2010) Export of microRNAs and microRNA-protective protein by mammalian cells. Nucleic Acids Res 38: 7248–7259. 20615901 10.1093/nar/gkq601 PMC2978372

[23] Chen X, Liang H, Zhang J, Zen K, Zhang CY (2012) Secreted microRNAs: a new form of intercellular communication. Trends Cell Biol 22: 125–132. 22260888 10.1016/j.tcb.2011.12.001

[24] Cortez MA, Bueso-Ramos C, Ferdin J, Lopez-Berestein G, Sood AK, et al (2011) MicroRNAs in body fluids–the mix of hormones and biomarkers. Nat Rev Clin Oncol 8: 467–477. 21647195 10.1038/nrclinonc.2011.76 PMC3423224

[25] Tan GS, Garchow BG, Liu X, Yeung J, Morris JPt, et al (2009) Expanded RNA-binding activities of mammalian Argonaute 2. Nucleic Acids Res 37: 7533–7545. 19808937 10.1093/nar/gkp812 PMC2794174

[26] Lima WF, Wu H, Nichols JG, Sun H, Murray HM, et al (2009) Binding and cleavage specificities of human Argonaute2. J Biol Chem 284: 26017–26028. 19625255 10.1074/jbc.M109.010835 PMC2758002

[27] Taganov KD, Boldin MP, Chang KJ, Baltimore D (2006) NF-kappaB-dependent induction of microRNA miR-146, an inhibitor targeted to signaling proteins of innate immune responses. Proc Natl Acad Sci U S A 103: 12481–12486. 16885212 10.1073/pnas.0605298103 PMC1567904

[28] Ceppi M, Pereira PM, Dunand-Sauthier I, Barras E, Reith W, et al (2009) MicroRNA-155 modulates the interleukin-1 signaling pathway in activated human monocyte-derived dendritic cells. Proc Natl Acad Sci U S A 106: 2735–2740. 19193853 10.1073/pnas.0811073106 PMC2650335

[29] Wang H, Zhang P, Chen W, Feng D, Jia Y, et al (2012) Evidence for serum miR-15a and miR-16 levels as biomarkers that distinguish sepsis from systemic inflammatory response syndrome in human subjects. Clin Chem Lab Med 50: 1423–1428. 22868808 10.1515/cclm-2011-0826

[30] Precone V, Stornaiuolo G, Amato A, Brancaccio G, Nardiello S, et al (2013) Different changes in mitochondrial apoptotic pathway in lymphocytes and granulocytes in cirrhotic patients with sepsis. Liver Int 16: 12169. 10.1111/liv.12169 23590253

[31] Vasilescu C, Rossi S, Shimizu M, Tudor S, Veronese A, et al (2009) MicroRNA fingerprints identify miR-150 as a plasma prognostic marker in patients with sepsis. PLoS One 4: 0007405. 10.1371/journal.pone.0007405 PMC2756627 19823581

[32] Wang JF, Yu ML, Yu G, Bian JJ, Deng XM, et al (2010) Serum miR-146a and miR-223 as potential new biomarkers for sepsis. Biochem Biophys Res Commun 394: 184–188. 20188071 10.1016/j.bbrc.2010.02.145

[33] Roderburg C, Luedde M, Vargas Cardenas D, Vucur M, Scholten D, et al (2013) Circulating microRNA-150 serum levels predict survival in patients with critical illness and sepsis. PLoS One 8: 23. 10.1371/journal.pone.0054612 PMC3555785 23372743

[34] Zhao H, Shen J, Medico L, Wang D, Ambrosone CB, et al (2010) A pilot study of circulating miRNAs as potential biomarkers of early stage breast cancer. PLoS ONE 5: e13735. 21060830 10.1371/journal.pone.0013735 PMC2966402

[35] Resnick KE, Alder H, Hagan JP, Richardson DL, Croce CM, et al (2009) The detection of differentially expressed microRNAs from the serum of ovarian cancer patients using a novel real-time PCR platform. Gynecol Oncol 112: 55–59. 18954897 10.1016/j.ygyno.2008.08.036

[36] Kirschner MB, Kao SC, Edelman JJ, Armstrong NJ, Vallely MP, et al (2011) Haemolysis during sample preparation alters microRNA content of plasma. PLoS One 6: 1. 10.1371/journal.pone.0024145 PMC3164711 21909417

[37] Cortez MA, Calin GA (2009) MicroRNA identification in plasma and serum: a new tool to diagnose and monitor diseases. Expert Opin Biol Ther 9: 703–711. 19426115 10.1517/14712590902932889

[38] Pickering MT, Stadler BM, Kowalik TF (2009) miR-17 and miR-20a temper an E2F1-induced G1 checkpoint to regulate cell cycle progression. Oncogene 28: 140–145. 18836483 10.1038/onc.2008.372 PMC2768269

[39] Bonauer A, Dimmeler S (2009) The microRNA-17–92 cluster: still a miRacle? Cell Cycle 8: 3866–3873. 19887902 10.4161/cc.8.23.9994

[40] Philippe L, Alsaleh G, Bahram S, Pfeffer S, Georgel P (2013) The miR-17 approximately 92 Cluster: A Key Player in the Control of Inflammation during Rheumatoid Arthritis. Front Immunol 4: 19. 23516027 10.3389/fimmu.2013.00070 PMC3601326

[41] Philippe L, Alsaleh G, Pichot A, Ostermann E, Zuber G, et al (2012) MiR-20a regulates ASK1 expression and TLR4-dependent cytokine release in rheumatoid fibroblast-like synoviocytes. Ann Rheum Dis 27: 27. 10.1136/annrheumdis-2012-201654 23087182

[42] Zhu D, Pan C, Li L, Bian Z, Lv Z, et al (2013) MicroRNA-17/20a/106a modulate macrophage inflammatory responses through targeting signal-regulatory protein alpha. J Allergy Clin Immunol 4: 00310–00312. 10.1016/j.jaci.2013.02.005 PMC5882493 23562609

[43] Zhu Y, Lu Y, Zhang Q, Liu JJ, Li TJ, et al (2012) MicroRNA-26a/b and their host genes cooperate to inhibit the G1/S transition by activating the pRb protein. Nucleic Acids Res 40: 4615–4625. 22210897 10.1093/nar/gkr1278 PMC3378857

[44] Witwer KW, Sisk JM, Gama L, Clements JE (2010) MicroRNA regulation of IFN-beta protein expression: rapid and sensitive modulation of the innate immune response. J Immunol 184: 2369–2376. 20130213 10.4049/jimmunol.0902712 PMC3076721

[45] Bakre A, Mitchell P, Coleman JK, Jones LP, Saavedra G, et al (2012) Respiratory syncytial virus modifies microRNAs regulating host genes that affect virus replication. J Gen Virol 93: 2346–2356. 22894925 10.1099/vir.0.044255-0 PMC3542124

[46] Min M, Peng LH, Sun G, Guo MZ, Qiu ZW, et al (2013) Aquaporin 8 expression is reduced and regulated by microRNAs in patients with ulcerative colitis. Chin Med J 126: 1532–1537. 23595390

[47] Wang JP, Hou XH (2007) Expression of aquaporin 8 in colonic epithelium with diarrhoea-predominant irritable bowel syndrome. Chin Med J 120: 313–316. 17374283

[48] Ding J, Huang S, Wang Y, Tian Q, Zha R, et al (2013) Genome-wide screening revealed that miR-195 targets the TNF-alpha/NF-kappaB pathway by downregulating IKKalpha and TAB3 in hepatocellular carcinoma. Hepatology 11: 26378. 10.1002/hep.26378 23487264

[49] Podolska A, Anthon C, Bak M, Tommerup N, Skovgaard K, et al (2012) Profiling microRNAs in lung tissue from pigs infected with Actinobacillus pleuropneumoniae. BMC Genomics 13: 1471–2164. 10.1186/1471-2164-13-459 PMC3465251 22953717

